# *CellBench*: *R/Bioconductor* software for comparing single-cell RNA-seq analysis methods

**DOI:** 10.1093/bioinformatics/btz889

**Published:** 2019-11-28

**Authors:** Shian Su, Luyi Tian, Xueyi Dong, Peter F Hickey, Saskia Freytag, Matthew E Ritchie

**Affiliations:** 1 Epigenetics and Development Division, The Walter and Eliza Hall Institute of Medical Research, Parkville, VIC 3052, Australia; 2 Department of Medical Biology, The University of Melbourne, Parkville, VIC 3010, Australia; 3 Epigenetics and Genomics, Harry Perkins Institute of Medical Research, Nedlands, WA 6009, Australia; 4 School of Mathematics and Statistics, The University of Melbourne, Parkville, VIC 3010, Australia

## Abstract

**Motivation:**

Bioinformatic analysis of single-cell gene expression data is a rapidly evolving field. Hundreds of bespoke methods have been developed in the past few years to deal with various aspects of single-cell analysis and consensus on the most appropriate methods to use under different settings is still emerging. Benchmarking the many methods is therefore of critical importance and since analysis of single-cell data usually involves multi-step pipelines, effective evaluation of pipelines involving different combinations of methods is required. Current benchmarks of single-cell methods are mostly implemented with *ad-hoc* code that is often difficult to reproduce or extend, and exhaustive manual coding of many combinations is infeasible in most instances. Therefore, new software is needed to manage pipeline benchmarking.

**Results:**

The *CellBench* R software facilitates method comparisons in either a task-centric or combinatorial way to allow pipelines of methods to be evaluated in an effective manner. *CellBench* automatically runs combinations of methods, provides facilities for measuring running time and delivers output in tabular form which is highly compatible with *tidyverse* R packages for summary and visualization. Our software has enabled comprehensive benchmarking of single-cell RNA-seq normalization, imputation, clustering, trajectory analysis and data integration methods using various performance metrics obtained from data with available ground truth. *CellBench* is also amenable to benchmarking other bioinformatics analysis tasks.

**Availability and implementation:**

Available from https://bioconductor.org/packages/CellBench.

## 1 Introduction

Single-cell transcriptome profiling offers researchers a powerful method for studying gene regulation at unprecedented resolution. Over the past 5 years, there has been a proliferation of specialized analysis algorithms for single-cell RNA-sequencing (scRNA-seq) data, including methods to deal with quality control, normalization, imputation, dimension reduction, clustering and trajectory analysis (Zappia *et al.*, 2018).

A typical scRNA-seq analysis workflow involves multiple interdependent steps, therefore it is important to benchmark not only individual methods, but also combinations of methods that form analysis pipelines to determine best practice in different settings. Subsequently, it is important that code written for benchmarking be extensible in order to be able to assess new tools or updates to existing methods that are frequently being released.

The *R/Bioconductor* (R Core Team, 2019; Huber *et al*., 2015) community has developed important infrastructure for single-cell data analysis. This includes the *SingleCellExperiment* object (Lun and Risso, 2019) for storing data and numerous packaged methods that are compatible with these objects for different types of scRNA-seq analysis. These packages form a comprehensive ecosystem for investigating various aspects of single-cell biology (Amezquita *et al.*, 2019).

Current scRNA-seq benchmarking efforts tend to focus on a particular analysis task, such as differential expression (Soneson and Robinson, 2018) or trajectory analysis (Saelens *et al*., 2019). Existing packages within Bioconductor that focus on methods comparisons are similarly task-centric and generally tailored for bulk RNA-seq, such as iCOBRA (Soneson and Robinson, 2016) for comparing differential expression analysis methods, and rnaseqcomp (Teng *et al*., 2016) for transcript quantification, while more general benchmarking software such as SummarizedBenchmark (Kimes and Reyes, 2019) does not facilitate efficient testing of combinations of methods. This led us to develop the *CellBench* software, which provides a framework to write structured benchmarking scripts, facilitates testing of combinations of methods and allows simple addition or removal of methods from pipeline steps.

## 2 Approach

**Fig. 1.**
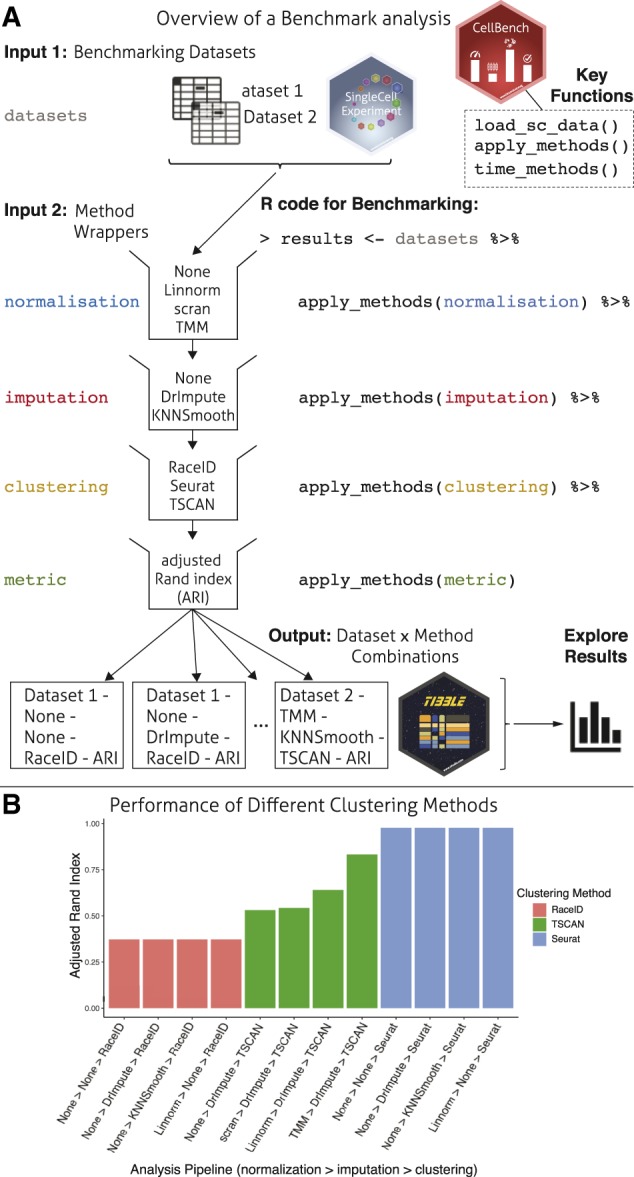
Schematic of a *CellBench* analysis. (**A**) Inputs to a benchmark analysis include data with known labels and collections of method wrappers that receive input and produce output in a consistent format. Methods that correspond to the same step in a pipeline are grouped into blocks, and the *CellBench* framework implicitly generates results for all combinations of methods. The code required reflects the diagram model and supports *piping* syntax. Results are returned in a *tibble* structure appropriate for manipulation with popular *tidyverse* packages (**B**) Using the *tibble* structure, the adjusted Rand index metric for combinations of normalization, imputation and clustering is filtered to plot results for the top 4 performing pipelines for RaceID, TSCAN and Seurat using ggplot2 (Wickham, 2016). The bar plot shows the relative performance of each clustering method and its sensitivity to upstream methods.

*CellBench* was developed to be simple to use and its design focussed on workflows with multiple steps, where each step has multiple candidate methods that may be applied, as shown in Figure 1A. This approach differs from existing Bioconductor methods by providing a generalised framework rather than focusing on specific analysis tasks, such as differential expression or clustering. The modular organisation of methods and automatic generation of combinations through chaining syntax allows for clear and reusable code (Fig. 1A).

The fundamental object in the *CellBench* framework is the *tibble* (Müller and Wickham, 2019), an extension of the standard *R data.frame* object with pretty printing features that makes it more compact and informative when displayed. Columns of the *tibble* identify the dataset and specific method run at each step, with the final column storing computational results from the specified combination. To extend the pipeline with additional steps, sets of methods are applied successively to the working *tibble* using the apply_methods function. This expands the number of rows to reflect new combinations, and updates the results column to contain new computational results. Under this framework, performance metrics used to compare the results from different algorithms are treated as a type of method to be applied to previous computational results. Examples of metrics include silhouette width, adjusted Rand index and number of clusters detected which can be compared to the ground truth available.

To use the apply_methods function, methods of the same pipeline step are stored as lists of functions in *R*. This creates a modular block (Fig. 1A) representing a specific step in a pipeline. The methods within a pipeline step are expected to take a common input type and produce a common output type, which allows new methods conforming to the input/output requirements to be added to the list, promoting collaboration and code reuse. In order to have methods accept the same type of input and produce the same type of output, wrapper functions will generally need to be written to perform some data manipulation before and after applying the core method. A vignette in the package introduces guidelines for writing effective wrappers as well as examples of pre-made wrappers.

*CellBench’s load_sc_data function provides* access to annotated single-cell datasets from a recent cross-platform control experiment with various experimental conditions and known cell-group identity (Tian *et al.*, 2019). Having access to processed and annotated datasets streamlines the benchmarking process to allow researchers to begin testing their methods more quickly and consistently. Additionally, *CellBench* offers convenience functions for sampling (sample_genes and sample_cells) and filtering (filter_zero_genes) *SingleCellExperiment* objects. Methods can be run in parallel to improve efficiency, errors are handled such that the overall benchmark can continue running even when individual pipeline combinations fail with errors and the time_methods function can be used to measure the running times of pipelines. Sub-sampling a *SingleCellExperiment* object using the sample_cells function allows run time to be measured on datasets of different sizes in a controlled manner.

To demonstrate application of *CellBench* in a multi-step benchmark, an example analysis is provided in a vignette. This analysis uses 2 datasets from Tian *et al*. (2019)(1 plate-based and 1 droplet based) and combines 4 normalization options (no normalization, *Linnorm* (Yip *et al*., 2017), *scran* (Lun *et al*., 2016) and *TMM* (Robinson and Oshlack, 2010)), 3 imputation options (no imputation, *DrImpute* (Gong *et al*., 2018) and *KNNSmooth* (Wagner, 2017)) and 3 clustering methods (*RaceID* (Grün *et al*., 2015), *Seurat* (Butler *et al*., 2018) and *TSCAN* (Ji and Ji, 2016)) as summarised in Figure 1A. For each of the 72 dataset × method combinations, the adjusted Rand index was used to measure the similarity between the clustering results obtained and the ground-truth available. Exploring the top four performing method combinations for each clustering algorithm on the plate-based dataset (Fig. 1B) allows researchers to compare their relative performance and sensitivity to upstream methods. A more comprehensive scRNA-seq benchmarking effort that used *CellBench* to compute 3,913 dataset × method combinations for tasks ranging from normalization, imputation, clustering, trajectory analysis and data integration was performed in Tian *et al*. (2019)with code available at https://github.com/LuyiTian/sc_mixology.

## 3 Discussion

*Ad-hoc* benchmarking scripts are error prone, difficult to share and difficult to extend with new methods. In existing benchmarking frameworks, individual combinations of methods need to be explicitly programmed, limiting the number of pipelines that can be feasibly compared. *CellBench* facilitates usage inside an interactive *R* session, and allows researchers to easily inspect data passing between pipeline stages. The fundamental objects of *tibbles* and *lists* are easy for a user to observe and manipulate, since they are already loaded in the environment. The ability to step through the code to observe intermediate results allows more effective debugging and faster prototyping compared to scripts that must be run from start to finish.

We have created a framework for researchers to evaluate the performance of different combinations of scRNA-seq analysis methods in a pipeline in a way that is reproducible and extensible. It leverages existing experience researchers have with the *R* programming language, as well as the popular *tidyverse* packages, facilitating software development and code sharing. Our focus on single-cell analysis has led to the development of a number of utility functions that are tailored for use with *SingleCellExperiment* objects. Although developed with scRNA-seq analysis in mind, *CellBench* can be easily used for benchmarking other bioinformatics analysis tasks. Future work will focus on deferring evaluation of pipelines such that combinations of pipelines can be set up and filtered down without being immediately evaluated. We also aim to develop a website that displays results from a versioned benchmark that will be updated over time to allow researchers to explore the rankings of different method combinations more fully.

*Conflict of Interest*: none declared.
